# Where and how have written action plans for atopic eczema/dermatitis been developed and evaluated? Systematic review

**DOI:** 10.1002/ski2.213

**Published:** 2023-03-22

**Authors:** Charankumal Singh Thandi, Sophie Constantinou, Rosie Vincent, Matthew J. Ridd

**Affiliations:** ^1^ Population Health Sciences Bristol Medical School University of Bristol Bristol UK; ^2^ Department of Dermatology Bristol Royal Infirmary Bristol UK

## Abstract

**Background:**

Atopic eczema/dermatitis is a common inflammatory condition which affects 15%–30% of children and 2%–10% of adults. It can have a significant impact and its management can be challenging. It is important for patients, parents, and caregivers to know how to look after their skin.

**Objectives:**

To identify and review written eczema action plans (WAPs) that are available internationally for use by patients, parents, and caregivers.

**Methods:**

We followed Preferred Reporting Items for Systematic reviews and Meta‐analysis guidelines. We searched relevant databases (MEDLINE, Embase, COCHRANE) from inception until March 2022. We sought grey literature via Google searches and professional networks. Database search results were independently reviewed by two different reviewers. With identified WAPs, we assessed length, appearance, content, how it was developed and whether it had been evaluated.

**Results:**

From 312 abstracts, supplemented by other searches, we identified 20 unique eczema WAPs. From nine countries, all were written in English with 18 were designed for children. For the majority, it was unclear whether any development work preceded their creation or the intended clinical setting for use. Nineteen had a stepwise approach, 17 advised when to seek help, 6 were visually appealing and 6 had a rationale behind treatment documented in the WAP. Only three had been evaluated in clinical trials.

**Conclusion:**

Further evaluation is needed to assess the effectiveness of the WAPs that currently exist, prior to creating further WAPs. Patient and caregiver involvement is needed in any future work.

## Graphical abstract

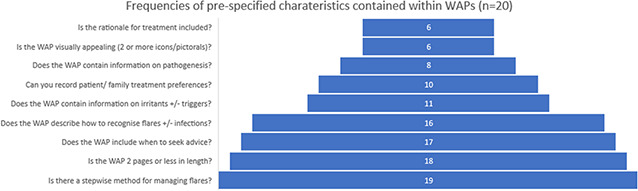

1

**What is known about this topic?**

- Atopic dermatitis or eczema is the most common childhood inflammatory skin condition affecting 15%–30% of children and 2%–10% of adults.
- Eczema written action plans (WAPs) serve as a record and reminder of treatment strategies, and their use is advocated by national guidelines to support self‐management

**What does this study add?**

- We have identified 20 unique WAPs, from 9 different countries and demonstrate that further evaluation of current WAPs is required to assess their effectiveness prior to creating new ones.

## 2 INTRODUCTION

Consultations for skin problems comprise around 1 in 7 primary care contacts.^1^ Atopic dermatitis or eczema is the most common childhood inflammatory skin condition affecting 15%–30% of children and 2%–10% of adults.^2^ Affected children suffer with significant impacts on quality of life, more so than diabetes and asthma.^3^ Depending on disease severity, treatments range from topical emollients and topical corticosteroids/calcineurin inhibitors (of different potencies), through to oral corticosteroids, systemic medications (such as ciclosporin and methotrexate)^4^ or biologic agents (such as dupilumab).^5^ Control rather than cure is the goal of treatment, which for many can be achieved with good adherence to prescribed therapies. Given the wide variety of topical treatments available and the impact of time consuming or ‘messy’ treatment regimens on quality of life, some parents and children can find it difficult to adhere to even relatively simple treatment regimens.^6^

Eczema written action plans (WAPs) serve as a record and reminder of treatment strategies, and their use is advocated by national guidelines to support self‐management.^7^ Although many children and adults will be prescribed similar topical treatments, their site, frequency, formulation or potency will differ. WAPs exist in many formats but essentially refer to a set of written instructions, which are individualised to the patient and state how to recognize and respond to changes. Used well, WAPs may improve treatment adherence and thereby improve outcomes for patients and, for children, their families.^8^

Powell et al.^9^ conducted a qualitative study which involved 41 semi‐structured interviews and two focus groups with parents of children with eczema, GPs and others clinicians and stakeholders. They found that participants felt positive about the role that WAPS could play with potential benefits of; a documented treatment plan, parent empowerment and improved clinical outcomes. Clinicians and caregivers identified useful aspects to include in an eczema WAP.

WAPs are commonly used for other conditions including asthma and viral induced wheeze^10^ for which there is more evidence on their effectiveness. A systematic review of 17 randomised control trials^11^ found that on asthma written actions plan reduced hospital admissions and presentations to the emergency department asthma. They reported that asthma WAPs with two or three action points were more effective than those with four points or more, suggesting that smaller, more concise amounts of information may lead to better outcomes. Agrawal et al.^12^ found that the addition of a written individualised home management plan improves the overall control of asthma in children with moderate persistent disease severity and should form an integral part of treatment protocols in addition to family education and pharmacological interventions. WAPs are routinely recommended in the clinical care of asthma^13^, ^14^ and annual reviews using WAPs are incentivised in general practice via the quality and outcome frameworks, which may also be used for eczema in a similar way in the future.

There are no published reviews of the number or types of eczema WAPs used internationally. Of the WAPs that are available, it is unknown how many were created using patient feedback, how many are validated and how effective they are. We sought to systematically identify and review when, where and how WAPs have been developed and evaluated.

## 3 MATERIALS AND METHODS

**FIGURE 1:**
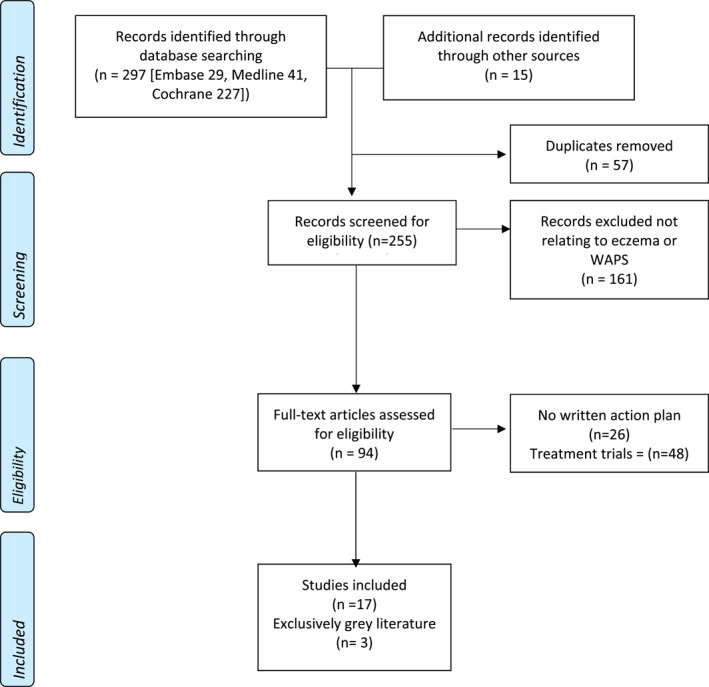
Preferred Reporting Items for Systematic reviews and Meta‐analysis diagram.

We followed Preferred Reporting Items for Systematic reviews and Meta‐analysis (PRISMA) guidelines^15^ and our PRISMA flowchart can be seen in Figure 1.

### 3.1 Information sources and search strategy

We searched MEDLINE, Embase, Cochrane from inception to 30 March 2022. In MEDLINE, we used the following strategy:

(Eczema/ or Dermatitis, Atopic/ OR eczematous dermatitides.tw OR eczematous dermatitis/tw or eczema*.tw OR atopic dermatitis.tw) AND (written action plan*.tw OR WAP*.tw OR eczema action plan*.tw OR eczema care plan*.tw OR atopic dermatitis action plan*.tw).

Searches for the other databases were matched as closely as possible to this using corresponding syntax and headings.

References and abstracts were downloaded into Endnote (Endnote X9, Thomson Reuters). All titles and abstracts were screened against the eligibility criteria by CST, RV and SC, independently verified by CST and SC. If there was any ambiguity from the title/abstract, the full paper was reviewed against the criteria. Additionally, grey literature was searched using Google in March 2022 and requests for information was sent via emails to specialist networks involving eczema research in July 2020 including the UKDCTN (UK Dermatology Clinical Trials Network), SAPC (Society for Academic Primary Care) Primary Care Dermatology Research Specialist Interest Group and ISAD (International Society of Atopic Dermatitis).

### 3.2 Eligibility criteria

We included WAPs for children (and/or their caregivers including parents) and adults with atopic eczema/atopic dermatitis, and papers that reported on their development and/or evaluation, not restricted by language.

### 3.3 Data extraction

Firstly, we sought to understand how the context for each WAP. This involved assessing where, how and for whom the WAP had been developed and evaluated. Secondly, we examined the format and content of each WAP, based on the criteria published by Powell et al. (Table 1).^9^ In the absence of any alternative framework, we felt this represented a valid checklist of criteria as it was developed with patient, caregiver and healthcare professional involvement. Thirdly, we sought information on how the WAP had been evaluated, both in terms of acceptability to users and effectiveness, such as improved patient knowledge, confidence or disease outcomes. CST led on data extraction focussing on whether each WAP addressed key areas, which were then independently verified by SC and RV, with adjudication by MJR in the event of any discrepancies.

**TABLE 1:** Checklist of criteria for data extraction from the WAPs from Powell et al.^9^

Is there a stepwise method for managing flares?
Does the WAP include when to seek advice?
Does the WAP contain information on eczema pathogenesis?
Is the rationale for treatment included?
Does the WAP contain information on potential irritants and/or triggers?
Does the WAP describe how to recognise flares and/or infections?
Is there an area in the WAP to record treatment preferences by the clinician?
Is the WAP length <2 pages in length?
Is the WAP visually appealing? (defined as containing 2 or more icons/pictorals)

### 3.4 Patient involvement

We worked with Eczema Outreach Society, who were running their own ecZema cAre Plan ‘ZAP’ project in parallel. Through a series of discussions, patients and caregivers identified what was valued and important to them, which informed our WAP assessment criteria and recommendations.

## 4 RESULTS

### 4.1 Eczema WAP development

We identified 20 WAPs (Table 2). Sixteen were published as papers between 2013 and 2019, the other four were identified through grey literature searches. Eighteen of the 20 WAPS were aimed at children, one was aimed at adults, and the remaining WAP did not specify a target age range. All were written in English. Eight were from the United States, three from New Zealand, two each from Australia, Canada, South Africa, United Kingdom and one each from Netherlands, Singapore and South Africa.

**TABLE 2:** Summary of eczema written action plans reviewed

Country	First author, year +/− plan name	1. Stepwise method for managing flares	2. When to seek advice	3. Information on pathogenesis	4. Rationale for treatment included	5. Information on irritants +/− triggers	6. How to recognise flares +/− infections	7. Record of treatment preferences	8. Two pages or less in length	9. Visually appealing	Count
UK	Powell et al., 2017^16^	•	•	•	•	•	•	•	−	•	8
UK	Rotherham hospital skin care plan^17^	−	−	•	•	−	−	−	−	−	2
USA	Chisolm et al., 2008^18^	•	•	•	•	•	•	•	•	−	8
USA	Boston Children's hospital WAP, Rork et al. 2012^19^	•	•	−	−	−	•	•	•	−	5
USA	Children's WAP, Shi et al., 2013^20^	•	•	•	•	•	•	−	•	−	7
USA	Adult's WAP, Shi et al., 2013^20^	•	•	•	•	•	•	−	•	−	7
USA	Rea et al., 2018^21^	•	•	−	−	•	•	•	•	•	7
USA	Brown et al., 2018^22^	•	•	•	−	−	•	•	•	−	6
USA	Brar et al., 2019^23^	•	•	−	−	−	•	−	•	−	4
USA	AAD, 2017^24^	•	•	−	−	−	•	−	•	−	4
Australia	ASCIA 2018^25^	•	•	−	−	•	•	−	•	−	5
Australia	Melbourne Children's hospital treatment plan^26^	•	−	−	−	−	−	•	•	−	3
Canada	Fernandes, 2017^27^	•	•	•	•	•	•	•	•	•	9
Canada	Eczema society of Canada WAP^28^ ^,^ ^a^	•	•	−	−	−	•	•	•	−	5
Netherlands	FIP Foundation^29^	•	•	•	−	•	•	−	•	•	7
Singapore	How et al. 2013^30^	•	•	−	−	•	•	•	•	−	6
South Africa	Allergy Foundation South Africa^31^	•	•	−	−	•	−	−	•	−	4
New Zealand	Kids Health Infected Eczema Plan^32^	•	•	−	−	−	•	•	•	−	5
New Zealand	Kids Health Eczema Care Plan^33^ ^,^ ^b^	•	•	−	−	•	•	−	•	•	6
New Zealand	Lyons et al. 2015^34^	•	−	−	−	−	−	−	•	•	3

Abbreviations: −, absent; •, present.

^a^ Eczema society of Canada WAP analysed no longer available, formerly here: https://www.eczemahelp.ca/eczema‐resources/.

^b^ Kids Health Eczema Care plan analysed has been subsequently updated, analysis based on original WAP.

Six WAPs were developed for use in a secondary care setting, two for use both in primary and secondary care, and one solely for use in primary care. However, in over half (11/20) of WAPs it was not clear whether they were intended for use in primary or secondary care. It was unclear whether any development work preceded the creation of 13 WAPs. Two groups stated they were based on pre‐existing asthma care plans^18^, ^22^ or other local resources.^25^ Three groups sought feedback from patients, caregivers and/or medical staff Brown.^19^, ^21^, ^29^ Powell et al.^16^ conducted semi‐structured interviews and held focus groups with parents of children with eczema, basing the content and design of the WAP around the preferences identified.

### 4.2 Eczema WAP format and content

**FIGURE 2:**
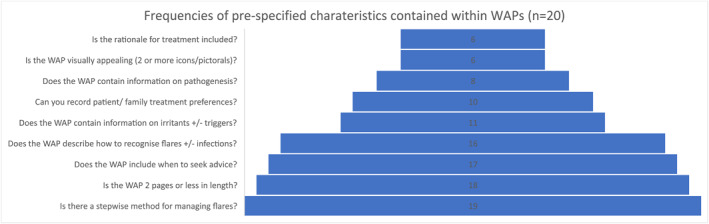
A summary of the information recorded in the written action plans.

19/20 WAPs contained stepwise information on managing flares, and 18/20 were two pages or less in length. The number of pre‐specified characteristics contained within each WAP is shown in Table 2 and Figure 2. Although most were aimed at children, only six were judged to be visually appealing. Eight contained information on eczema pathogenesis and six on the rationale for treatment strategy. Most (17/20) contained advice on when to seek help from healthcare professionals.

### 4.3 Eczema WAP effectiveness

The effectiveness of only three eWAPS had been evaluated in a clinical trial. Duhovic et al., comparing WAP versus verbal instruction, found no benefit.^35^ Shi et al. found a statistically significant improvement in atopic dermatitis recognition, management and prevention in the WAP group compared to those with just written information on eczema.^20^ Rea et al., comparing a paediatric WAP with conventional care, found a non‐statistically significant improvement in eczema control in the group receiving the WAP. However, 62% had previously been provided with a WAP by their care providers.^21^

## 5 DISCUSSION

This study demonstrates that the published literature on eczema WAPs is limited. The 20 WAPs reviewed have been developed with different levels of patient input, in different countries and designed for use in different care settings. They are of varying quality, contain different amounts of information and most have not been evaluated. The majority of WAPs have been developed in North America for use by carers of children, without any published justification for their design or content. Of the 20 WAPs identified, only 3 have described seeking feedback from patients.^19^, ^21^, ^29^ There is limited evidence from a trial of one eWAP that it may improve patient understanding.^20^

We present a prospectively registered systematic review, with a comprehensive literature search in large medical literature databases, supplement by with efforts to identify unpublished and grey literature. All screening and data extraction was done independently by at least two authors, with data collected and presented in line with PRISMA guidance. We evaluated WAPs using specified characteristics published by Powell et al.^9^ which were generated through qualitative work with patients and stakeholders. Internationally, it is likely some WAPs may be used in clinical practice without publication, and therefore may not have been included in our search. Additionally, grey literature searches were limited to English. One WAP analysed is no longer available^28^ and one has been recently updated to include pictorals.^33^ The database searches are, however, likely to have captured all published WAPs as we purposely kept our search criteria broad, consulting with an information specialist on correct and appropriate search terms. Alternative ways of assessing the format and content of the WAPs other than Powell et al.^16^ may have drawn different conclusions and while we counted the number of features present, this does not infer greater potential effectiveness.

This review highlights the need for further evaluation of existing WAPs, rather than the creation of new ones. This may include translation or back‐translation and validation of eczema WAPs into languages other than English. Although WAPs that featured pictures or icons aimed were primarily aimed at children, including pictorals may be beneficial to all parents, especially adults with poor (health) literacy skills. Indeed, most WAPs were targetted at children, and further work to develop and evaluate WAPs targetted at adults with eczema is needed. None of the HOME recommended outcome instruments^36^ were used in the two trials identified, which we recommend be included in future RCTs.

It remains difficult to make recommendations on which criteria should be included in WAPs. The WAPs by Chilsolm et al.,^18^ Powell et al.^16^ and Fernandes^27^ include the highest number of categories, but only the WAP created by Shi et al. shows evidence of effectiveness.^20^ We provide links in the references, where available, to the WAPs so that clinicians can use this review to decide which best suits their setting and patient population, with the highest scoring three identified above.

## COMMENT ON FINDINGS FROM PATIENT AND PUBLIC CONTRIBUTOR (MAGALI REDDING, OF THE CHARITY ECZEMA OUTREACH SUPPORT)

By supporting families of children with eczema for over 10 years, Eczema Outreach Support has gained deep insights into the issues faced by its beneficiaries. The lack and inconsistency of written eczema care plans is one of the most damaging ones. Parents and carers often go home after their clinic appointment without having fully understood their child's treatment and with no clear guidance to refer to. This dramatically limits their ability to self‐manage at home, increases the likelihood of avoidable repeat appointments, and most importantly negatively impacts on their child's long‐term health outcomes. Other families may be lucky enough to have been given a quality written action plan, however this will entirely depend on their clinician's access to such resource. Moreover, as highlighted in this review, it is not clear how these plans are developed and assessed, if at all. There is therefore an urgent need to design and evaluate a self‐management resource for families with eczema. A quality improvement partnership between clinicians, academics and families has the potential to result in a gold standard eczema written plan which could change the lives of people with eczema and their families across the UK and become a model for other conditions.

## CONFLICT OF INTEREST

MJR developed the eczema Written Action Plan (eWAP) criteria and tool, included in this review.

## AUTHOR CONTRIBUTIONS

**Charankumal Singh Thandi**: Conceptualization (Equal), Data curation (Equal), Formal analysis (Equal), Funding acquisition (Equal), Investigation (Equal), Methodology (Equal), Writing – original draft (Equal), Writing – review & editing (Equal). **Rosie Vincent**: Data curation (Equal), Formal analysis (Equal), Methodology (Equal), Writing – original draft (Equal), Writing – review & editing (Equal). **Sophie Constantinou**: Data curation (Equal), Formal analysis (Equal), Writing – original draft (Equal), Writing – review & editing (Equal). **Matthew J. Ridd**: Conceptualization (Equal), Data curation (Equal), Formal analysis (Equal), Investigation (Equal), Methodology (Equal), Supervision (Equal), Writing – original draft (Equal), Writing – review & editing (Equal).

## ETHICS STATEMENT

Not applicable.

## Data Availability

The data that support the findings of this study are openly available by accessing our references in the paper.
